# Knowledge, Attitudes and Practices Related to Visceral Leishmaniasis in Rural Communities of Amhara State: A Longitudinal Study in Northwest Ethiopia

**DOI:** 10.1371/journal.pntd.0002799

**Published:** 2014-04-17

**Authors:** Noemí López-Perea, Luis Sordo, Endalamaw Gadisa, Israel Cruz, Tsegaye Hailu, Javier Moreno, Abraham Aseffa, Carmen Cañavate, Estefanía Custodio

**Affiliations:** 1 Centro Nacional de Epidemiología, Instituto de Salud Carlos III, Madrid, Spain; 2 CIBER en Epidemiología y Salud Pública (CIBERESP), Departamento de Medicina Preventiva y Salud Pública, Facultad de Medicina, Universidad Complutense de Madrid, Madrid, Spain; 3 Armauer Hansen Research Institute, Addis Ababa, Ethiopia; 4 WHO Collaborating Center for Leishmaniasis, Servicio de Parasitología, Centro Nacional de Microbiología, Instituto de Salud Carlos III, Madrid, Spain; 5 Centro Nacional de Medicina Tropical, Instituto de Salud Carlos III, Madrid, Spain; Independent Consultant, Spain

## Abstract

**Background:**

In the northwest of Ethiopia, at the South Gondar region, there was a visceral leishmaniasis (VL) outbreak in 2005, making the disease a public health concern for the regional health authorities ever since. The knowledge on how the population perceives the disease is essential in order to propose successful control strategies.

**Methodology/Principal findings:**

Two surveys on VL knowledge, attitudes and practices were conducted at the beginning (May 2009) and at the end (February 2011) of a VL longitudinal study carried out in rural communities of Libo Kemkem and Fogera, two districts of the Amhara Regional State. Results showed that VL global knowledge was very low in the area, and that it improved substantially in the period studied. Specifically, from 2009 to 2011, the frequency of proper knowledge regarding VL signs and symptoms increased from 47% to 71% (p<0.0001), knowledge of VL causes increased from 8% to 25% (p<0.0001), and knowledge on VL protection measures from 16% to 55% (p<0.0001). Moreover, the improvement observed in VL knowledge was more marked among the families with no previous history of VL case. Finally, in 2011 more than 90% of the households owned at least an impregnated bed net and had been sprayed, and attitudes towards these and other protective measures were very positive (over 94% acceptance for all of them).

**Conclusions/Significance:**

In 2009 the level of knowledge regarding VL was very low among the rural population of this area, although it improved substantially in the study period, probably due to the contribution of many actors in the area. VL patients and relatives should be appropriately informed and trained as they may act as successful health community agents. VL risk behavioural patterns are subject to change as attitudes towards protective measures were very positive overall.

## Author Summary

Visceral leishmaniasis (VL) is a vector borne disease that can be fatal if left untreated. In northern Ethiopia there was a VL outbreak in 2005, making the disease a public health challenge ever since. In order to promote the participation of communities in the control of the disease, it is essential to know how they perceive the disease and its management. There is a paucity of studies dealing with the knowledge, attitudes and practices (KAP) towards VL in the world in general and in rural Ethiopia in particular. We conducted two KAP studies at the beginning and at the end of a VL longitudinal study carried out between 2009 and 2011. The project included VL community talks and sensitization, and there were other interventions implemented by different actors in this period. Our results showed that, among the rural communities surveyed, the knowledge regarding signs and symptoms, causes, and protective measures of the disease was very low. However, it improved substantially in the period studied, suggesting that knowledge was subject to change by community interventions. It also showed that VL patients and relatives can act as successful health agents and that the population had positive attitudes towards the implementation of preventive actions.

## Introduction

Visceral leishmaniasis (VL) (also known as kala-azar) is a vector-borne neglected disease caused by the protozoan parasite *Leishmania donovani* in East Africa, and transmitted by the bite of female phlebotomine sand fly. Clinical signs and symptoms often include long lasting and irregular fever, weight loss and hepato-splenomegaly; and it is fatal if left untreated [1]. More than 90% of global VL cases occur in six countries: India, Bangladesh, Sudan, South Sudan, Ethiopia and Brazil. Globally, 200,000 to 400,000 new cases of VL occur every year, and only in Ethiopia it is estimated an annual incidence of 4,000 new cases [2]. The principal foci in Ethiopia are the one in the Northwest border with Sudan (Metema and Humera), and the one located in the South, in the Segen and Woito river valleys [3]–[6].

In Libo Kemkem and Fogera (highland districts in South Gondar, Amhara Regional State) VL had never been reported until May 2005 when a large VL outbreak was identified, with more than 2,500 cases treated. A high mortality rate was reported initially, probably due to the long time required for the recognition of the epidemic [7]. Migration of laborers coming from endemic neighboring areas (border of Sudan) is one of the hypotheses for the introduction of VL in the region [8], [9] that has become a public health concern for the Amhara Regional State Health Bureau ever since.

In order to elaborate successful VL control programs it is essential to know the risk factors associated with it, and to understand the disease-related knowledge, attitudes, and practices (KAP) of the population [10]. The factors associated with *Leishmahia* infection in this area have already been described, being related to past history of VL in the household, house conditions or behaviors like sleeping outside, among others [11]. The factors associated with the VL clinical manifestation in this area were sleeping outside or under an acacia tree were among others [8].

However, little is known about how individuals in rural communities of this region perceive the disease and its management. There is a paucity of VL KAP studies in the New World [12], [13] and in the Old World [14]–[18] in general. And in Ethiopia, to the best of our knowledge, there are no published studies that have focused on these aspects in a rural setting. Only recently it has been published a VL KAP study conducted in Addis Zemen, the urban centre of Libo Kemkem [19]. We expect our study, focused in the rural, to contribute to those urban results, in order to help the Amhara health authorities to promote the involvement of the communities in the control of the disease, a priority for the government of Ethiopia [20].

Health education campaigns should be adapted, in contents, type, and format to the target population [1], [21]. In other settings it has already been proven that educational strategies with informative materials can contribute to VL control programs [22]–[24], but written materials in rural communities of Ethiopia with high levels of illiteracy may not be appropriate. In the area of study, since the 2005 outbreak, there have been different actors implementing outreach activities with health education and case screening. And the research study that we conducted included informative and sensitization talks, which may be more appropriate for this population. By carrying out two KAP surveys at the beginning and at the end of the longitudinal study we look forward to assess baseline VL knowledge attitudes and practices, as well as the change in VL knowledge along the study period.

Furthermore, as results from other *Leishmaniasis* KAP studies have suggested that the knowledge of the disease is restricted to those that have suffered from it personally or in a person closely related [25], we wanted to differentiate the results regarding VL knowledge by households with and without a positive history of VL.

Therefore, the aims of this study are 1) to assess the knowledge, attitudes and practices of VL in households of a rural endemic area of Amhara Regional State, Ethiopia and 2) to evaluate the impact of community interventions in the VL knowledge at household level between 2009 and 2011, taking into account the previous VL history of the participant households.

## Materials and Methods

### Ethics statement

The study was approved by the ethical advisory boards of the Armauer Hansen Research Institute and the Ethiopian National Ethical Committee in Ethiopia, and the Instituto de Salud Carlos III in Spain. Support letters were obtained from the Amhara Regional State Health Bureau and the different districts' Health Offices. All parents/guardians gave written informed consent prior to responding to the questionnaires directed to them and to the enrolment of their children in the study, and assent was also obtained from children ≥11 years of age.

### Study area and population

The area of study was located in the Amhara Regional State, South Gondar, Northwest Ethiopia (See Custodio *et al.* [11] for geographical location of study site), and comprised two districts (*weredas*) mainly rural: Libo Kemkem (being Addis Zemen town its capital) and Fogera (being Woreta town its capital). These are adjacent districts most affected by the outbreak of VL occurred in 2004–2005 [26]. According to year 2009 census, the population was 198,374 and 226,595 for Libo Kemkem and Fogera respectively.

### Study design

The KAP surveys presented in this work were carried out within the framework of a prospective longitudinal study entitled “Visceral Leishmaniasis and Malnutrition in Amhara State, Ethiopia”. The study involved a cross sectional survey conducted in May 2009 to estimate the prevalence and associated factors of VL and malnutrition in school-aged children, and a cohort study that was carried out between May 2009 and February 2011 in order to elucidate the relationships between malnutrition and *Leishmania* infection in this same age group. The study consisted of four surveys that were carried out in May 2009, December 2009, May 2010 and February 2011. In the first and last surveys questions related to the knowledge, attitudes and practices (KAP) towards visceral leishmaniasis and *Leishmania* transmission were addressed to the care providers (present at the household at the time of the survey) of the children participant in the cohorts' study.

Population sampling was carried out by multi-staged cluster survey being the primary sampling units the sub-districts (*kebeles*) with high incidence of VL: Bura, Yifag Akababi, and Agita from Libo Kemkem; and Sifatra and Rib Gebreal from Fogera. Secondary sampling units were randomly selected villages (*gotts*) in each of the selected sub-districts, and third sampling units randomly selected households in each of the villages. The sample size was calculated according to the objective of the original project, described in detail elsewhere [9], [11], [27].

### Data collection

In May 2009 the care providers of the children recruited for the cohort study were interviewed by trained local personnel using a standardized structured questionnaire that included questions on demographics, household characteristics, child health, VL risk factors and VL KAP. A question regarding if someone in the household had suffered VL in the past was included in order to elaborate the variable Household (HH) with positive history of VL. This variable was based on the interviewee's report, but not verified by treatment or medical record. The use and the number of bed nets owned by the household was also reported but not verified by the interviewer.

In February 2011 the same households were visited, and the interview consisted of a standardized questionnaire that included questions related to child health and a more extensive VL KAP. However, the question regarding if someone in the household had suffered VL in the past was not included in this last interview. Care providers present in the house at the time of this visit were not necessarily the same who were interviewed in the first survey (only 40% of them were the same person in the May 2009 and in the February 2011 surveys). Therefore, we assess knowledge at household level and not at the individual one.

Out of the 276 households visited in May 2009 we were able to collect data on 218 when revisited in February 2011, which, for the purpose of this study, were the ones to be kept in the analysis.

All questionnaires were translated in to Amharic, the main local language.

### Variables

The outcome variables regarding *“Awareness”* were based on self-perceived knowledge related to VL sings and symptoms, causes, or protective measures respectively (as an example: *Do you know any sign or symptom of VL? Yes/No//If yes, which ones?)*. The different answers were thereafter converted into dichotomous variables.

And the outcome variables regarding *“Proper knowledge”* were created as follows:

- Proper knowledge of VL sings/symptoms: a spontaneous answer including at least one of the following: “Fever”, “Abdominal swelling” or “Weight loss”.
- Proper knowledge of VL causes: a spontaneous answer including insect or sand fly (included in the table as “Insect”).
- Proper knowledge of VL protective measures: a spontaneous answer that included at least one of the following “Bed net”, “Environmental sanitation”, “Spraying” or “Avoiding acacia tree”.

### Description of the intervention

Before the starting of the project, a two days consultation was made with the local community leaders, sub-district (*kebele*), and district administrators in order to approach why the project was relevant, what was the VL situation in the area, and also to cover VL general information.

During the cohorts study four surveys were conducted. The day before visiting the community for the first data collection (May 2009 survey), the supervisor together with the *kebele* administrator and the community leader conducted a one day sensitization talk to every elder in the community. And before each of the following surveys the supervisor talked to the household head or adult present in the house at the time of the visit. The talks covered VL general signs and symptoms as well as *Leishmania* infection ways of transmission and protective measures. In addition, in January 2011 a special informative meeting was held with the community leaders of all participant *gotts* and with *kebeles* administrator in order to promote leader's encouraging to families to participate in the fourth and last survey of the project.

### Data management and analysis

During the data collection process questionnaires were checked on site by the supervisor and, once they were completed, were submitted to the data processing unit of the Armauer Hansen Research Institute (AHRI) in Addis Ababa, Ethiopia, where they were double entered in ACCESS and cross checked for consistency. Joint data analysis was conducted in the Spanish National Centre of Tropical Medicine in Madrid, Spain, where data was rechecked and cleaned. Finally, data analysis was performed using STATA version 11 (Stata Corp., College Station, TX, USA).

Descriptive statistics were performed and the Chi square test was used for comparisons between HH with and without positive history of VL, except when the number in any of the categories analysed was below 5, that the Fisher's exact test was applied.

Differences in results pre (2009) and post (2011) implementation of the study were examined by the McNemar test for matched data.

All *p*-values were two tailed and a *p*-value of ≤0.05 was taken as significant.

## Results

A total of 218 households were surveyed, all of them from rural environment with uniform low socioeconomic conditions, described in **Table 1**. In 2009, the majority of the heads of the households were male (91.3%), illiterate (78%) and had a principal occupation related to the cultivation of land (99.8%). Among those who had their own lands (97.7%) the mean of acreage owned was 1.2 Ha (SD: 6.7) and only 11% had more than 3 Ha. The mean household size was 6.1 persons (SD: 1.7), with households size ranging from 3 to 10 persons. Radios were present only in 28% of the households.

**Table 1:** Sample households characteristics (N = 218).*

Characteristic	Categories	Number†	%
**Sex of head of household**	**Male**	199	91.3
****	**Female**	19	8.7
**Age of head of household (years)**	**<34 years**	36	16.7
****	**35–44 years**	89	41.2
	**≥45 years**	91	42.1
	**Illiterate**	157	78.1
**Head of household's years of education**	**1–4 years**	22	10.9
****	**5–9 years**	19	9.5
	**10+ years**	3	1.5
**Total members in the household**	**<6**	132	60.6
	**7–9**	75	34.4
	**≥10**	11	5.0
**Land owned by the household (Has)**	**0–1**	142	67.0
****	**2–3**	46	21.7
	**>3**	24	11.3
**Total crops produced in the household per year (Kgs)**	**0–400**	61	28.0
****	**401–800**	71	32.6
****	**>800**	86	39.4

*Information only collected in the 2009 survey.

† Totals may not add up to 218 due to missing values.

The main source of knowledge regarding VL in 2009, before the implementation of the study, was the health centre (n = 57, 26.1%) followed by knowing someone that had suffered VL (n = 21, 9.6%).

In 2009, 47.7% of the population surveyed reported to be aware of VL signs and symptoms, versus an 84.7% in 2011 (p<0.0001). The most frequently reported signs and symptoms were abdominal swelling, fever, weight loss, and low appetite. Furthermore, in 2011 a significantly higher frequency of interviewees had “Proper knowledge of VL signs and symptoms” as compared to 2009 (71.1% to 14.6%, p = 0.0001). And, when proper knowledge on VL signs and symptoms was stratified by the variable if someone in the household had suffered VL in the past, a higher proportion of respondents living in houses with past history of VL reported correct signs and symptoms, being this difference more marked in 2009 (94% to 28%, p<0.0001) than in 2011 (89% to 64%, p = 0.001) (**Table 2**).

**Table 2:** Knowledge about VL signs and symptoms.

		2009						2011						
Item	Categories	Total (218)		HH with history of VL*				Total (218)		HH with history of VL*				OR (CI95%)† 2011–2009
		N	%	Yes (63)		No (155)		N	%	Yes (63)		No (155)		
				n	%	n	%			n	%	n	%	
**Awareness of VL signs and symptoms**	**Yes**	**104**	**47.7**	61	96.8	43	27.7§	**172**	**84.7**	60	98.4	112	80§	**16.4 (6.7–51.8)**
	Abdominal swelling	77	35.3	41	65.0	36	23.2§	117	53.7	38	63,3	79	51	2.1 (1.4–3.3)
	Fever	67	30.7	44	69.8	23	14.8§	70	32.1	36	57.1	34	21.9§	1.1 (0.7–1.8)
	Weight loss	28	12.8	21	33.3	7	4.5§	42	19.3	16	25.4	26	16.8	1.7 (0.9–3.1)
	Low appetite	25	11.5	19	30.2	6	3.9§	32	14.7	17	27	15	9.7§	1.4 (0.7–2.7)
**VL signs and symptoms**	Foot edema	14	6.4	11	17.5	3	1.9§	10	4.6	4	6.3	6	3.9	0.7 (0.3–1.7)
	Face edema	6	2.7	4	6.4	2	1.3	14	6.4	4	6.3	10	6.5	2.3 (0.8–7.4)
	Diarrhea	4	1.8	2	3.2	2	1.3	17	7.8	6	9.5	11	7.1	4.3 (1.4–17.4)
	Epistaxis	11	5.0	6	9.2	5	3.2	21	9.6	9	14.3	12	7.7	2.0 (0.9–4.8)
	Other‡	5	2.3	1	1.6	4	2.6	17	7.8	7	11.1	10	6.5	4.0 (1.3–16.4)
**Proper knowledge of VL signs/symptoms** ¶	**Yes**	**102**	**46.8**	**59**	**93.6**	**43**	**27.7** §	**155**	**71.1**	**56**	**88.9**	**99**	**63.8** §	**3.5 (2.1–6.0)**

Results for 2009 and 2011 surveys, stratified by households with and without history of VL.

(*) As reported in the 2009 survey.

(†)Results of McNemar test for matched data.

(§)p<0,05 for differences between HH with and without history of VL.

(‡)Other signs and symptoms reported: foot and face edema, epistaxis, fatigue, chills, headache and vomiting.

(¶)Proper knowledge defined as a spontaneous answer that included at least one of the following “Fever”, “Weight loss” or “Abdominal swelling” in the VL signs & symptoms question.

Regarding self-perceived knowledge of the possible causes of the disease, a 16.5% reported to be aware of VL causes in 2009 versus a 58.7% in 2011 (p<0.0001). The answer considered appropriate, “Insect”, was the one most frequently reported in 2009 (8.3%) and increased to 31.8% in 2011 (p<0.0001). Respondents living in houses with past history of VL reported more frequently a proper knowledge on the vector borne disease nature of VL than respondents living in houses with no history of VL, although this difference was only significant in 2009 (p<0.001)(**Table 3**).

**Table 3:** Knowledge about VL causes and protection measures.

		2009						2011						
Item	Categories	Total (218)		HH with history of VL*				Total (218)		HH with history of VL*				
		N	%	Yes (63)		No (155)		N	%	Yes (63)		No (155)		OR (CI95%)† 2011–2009
				n	%	n	%			n	%	n	%	
**Awareness of VL causes**	**Yes**	**36**	**16.5**	**23**	**36.5**	**13**	**8.4** §	**128**	**58.7**	**45**	**71.4**	**83**	**53.5** §	**11.3 (5.7–25.5)**
	Acacia tree	16	7.3	10	15.9	6	3.9§	84	38.5	28	44.4	56	36.1	10.7 (4.9–27.6)
**VL Causes**	Insect**	18	8.3	13	20.6	5	3.2§	55	25.2	20	31.7	33	21.3	4.5 (2.2–10.0)
	Other‡	6	2.7	2	3.2	4	2.6	22	10.1	6	9.5	16	10.3	3.7(1.4–11.1)
**Awareness of VL protection measures**	**Yes**	**45**	**20.6**	**26**	**41.3**	**19**	**12.3** §	**124**	**57.9**	**41**	**65.1**	**83**	**55.0**	**5.6 (3.3. 10.1)**
	Bed-net	27	12.4	18	28.6	9	5.8§	105	48.2	36	57.1	69	44.5	8.1 (4.3–16.8)
**VL protection measures**	Environmental sanitation	12	5.5	5	7.9	7	4.5	30	13.8	8	12.7	22	14.2	3.0 (1.4–7.3)
	Other¶	16	7.3	9	14.3	7	4.5§	15	6.9	6	9.5	9	6.7	0.9 (0.4–2.0)
**Proper knowledge of VL protective measures** ††	**Yes**	**35**	**16**	**21**	**33.3**	**14**	**9** §	**119**	**54.6**	**39**	**61.9**	**80**	**51.6**	**7.9 (4.3–15.9)**

Results for 2009 and 2011 surveys stratified by households with and without VL history.

(*) As reported in the 2009 survey.

(†)Results of McNemar test for matched data.

(**) Encompasses “Insect” and “Sand flies” answers, and is the only response considered as “Proper knowledge on VL causes”.

(§)p<0.05 for differences between HH with and without history of VL.

(‡)Other causes reported were poor hygiene, malnutrition, hunger, polluted water and sexual intercourse with infected person.

(¶)Other protective measures reported were spraying, treatment, good nutrition and avoiding acacia trees.

(††)Proper knowledge on VL measures defined as a spontaneous answer that included at least one of the following “Bed net “,“Environmental Sanitation”, “Spraying” or “Avoiding acacia tree” in the VL protective measures question.

In relation to VL protection measures, in 2009 only 21% of the respondents declared to be aware of how to protect from VL, in regard to 58% in 2011, p<0.0001. The most mentioned protection measures in both years were “Bed Nets” and “Environmental Sanitation”, but the probability of giving a correct answer regarding VL protective measures was almost 8 times higher in 2011 than in 2009 (p<0.0001). When stratified by houses with and without VL history, differences were found only in the responses of the 2009 survey, were proper knowledge on VL protective measures was reported more frequently among respondents of houses with positive history of VL (33.3% versus 9.0%, p<0.001) (**Table 3**).

In relation to attitudes and practices, in 2009 57% of the houses reported to own bed nets versus a 98% in 2011, p<0.0001. The only reason given for not owning bed nets in 2011 was “Because it is difficult to get them”. The number of households owning two or more bed nets increased from 56 (25.7%) in 2009 to 177 (81.2%) in 2011 (**Table 4**). In the majority of the houses, (n = 181, 85%) respondents reported that bed nets were used by all members in the family, followed by the option “Only adults” (n = 14, 6%) and “Mother and children” (n = 7, 3%). Moreover, 94% of respondents stated that they would accept using impregnated bed nets in the house.

**Table 4:** Practices related to household characteristics and protection measures associated with *Leishmania* transmission in 2009 and in 2011.

Item	Categories	2009		2011		OR (CI95%)†
		N	%	N	%	2011–2009
**Bed nets**						
**Are there bed nets in the HH?**	Yes	124	56.9	213	97.7	90 (15.7–3593.8)
**How many bed nets are there in the HH?**	One	68	31.2	34	15.6	0.4 (0.2–0.7)
	Two	54	24.8	143	65.6	5.2 (3.3–8.8)
	Three	1	0.4	34	15.6	34 (5.7–1381.9)
**Household roof**						
**House roof material**	Corrugated iron	134	61.8	143	65.6	2.8 (1.0–9.9)
	Straw	83	38.2	74	33.9	0.3 (0.07–1.0)
**Spraying**						
**Has your house ever been sprayed?**	Yes	140	64.2	209	95.9	18 (6.7–67.8)

(†)Results of McNemar test for matched data.

In 2011 there were 143 (66%) houses with iron roof, an increase from the 134 (62%) in 2009 but no significant (p = 0.06) (**Table 4**). The main reasons reported for using iron roof were because it was more solid (n = 60, 40%) and better for weather conditions (n = 51, 36%). The reason for using straw (n = 74, 33%), the alternative roof material, was its lower price (n = 57, 77%). In relation to house conditions, more than 90% of the houses surveyed (n = 198) had cracks in the wall, and when the interviewees were asked about the optimal frequency for repairing them, the responses ranged from “Never” (n = 15, 6.9%), “Every 2 years or more” (n = 32, 15%), “Once a year” (n = 56, 26%), “More than once a year” (n = 79, 36%), to “Every month” (n = 32, 15%).

In 2011 almost every surveyed house (96%) had been sprayed compared to 64% in 2009 (p<0.0001) (**Table 4**). The acceptance for indoor and outdoor spraying was very high (98% to 97% respectively). And so it was the acceptance for house surroundings environmental cleaning (99%).

Of the houses surveyed, in 2011, 63% (n = 138) reported having members of the family sleeping outside, mainly due to far away herding or cattle watching in the house surroundings. However, 29 interviewees (21%) reported that family members sleeping outside made use of bed nets, and 20 (15%), declared that other protection measures like cloths, blankets or environmental sanitation were used. The reasons reported for not using bed nets while sleeping outside ranged from “Bed nets are difficult to use when sleeping outside” (40%), “Lack/shortage of bed nets” (11%) to “Bed nets are too expensive” (11%). Finally, more than 80% (n = 174) of respondents declared that at least one member of the family rested under acacia tree, a risk factor for VL in the area, and the time of the day most frequently reported for doing it was during midday (n = 144, 83%).

In 2011, the first option for VL treatment was public health facilities (n = 215, 99%), and only 3 persons (1.3%) mentioned home remedies or traditional healer as first choices, based on “Better to try home first” and “Fear of evil eye” respectively.

## Discussion

The present study shows that knowledge regarding visceral leishmaniasis in the rural communities of this region of Ethiopia is low, although it improved substantially among the households participating in the longitudinal research project described before, that was carried out between 2009 and 2011. The improvement in knowledge was substantially more marked among the families with no past history of VL.

This study was focused in rural population because in Africa VL is mainly transmitted in rural settings [28] and, as stated in the objectives of the Sixtieth World Health Assembly of 2007, one of the means to combat leishmaniasis is to improve knowledge about, and skills to prevent, the disease among people in rural areas [29].

In the survey conducted in May 2009, before the implementation of the study, the level of awareness related to VL signs and symptoms was around 50%, substantially lower than results from other rural communities of Nepal and India where it raises up to 85% [18], [30], [31]. This may be due to the fact that VL has been endemic for more than 20 years in the settings of the Indian subcontinent studied, and in Libo Kemkem and Fogera districts the disease has only recently been known, at least as a public health problem [26]. However, the level of awareness was also significantly lower than the one found in the urban population of this same area that reached 83%. Furthermore, in this urban population 62% of interviewees were able to identify more than one VL symptom [19], whereas only 47% were able to report at least one correct symptom in the rural population of our study. This could be explained by the location of the VL treatment center in Addis Zemen town, which allowed neighboring communities to get information and education related to the disease. And also by the higher level of illiteracy in the rural communities (72% compared to the 34% found in the Addis Zemen town study), that has been associated with poorer VL knowledge before [31]. Future education activities should be aimed to making rural population more knowledgeable of the symptoms of the disease, as proper perception prompts patients to seek early treatment, essential in VL cure and complete recovery [18].

Awareness of VL causes was as low as 16% in 2009, and correct knowledge of the vector-borne character of VL was also remarkably low (8% of respondents mentioned mosquitos and only 1 respondent specifically sand flies) compared to rural communities in other countries, where high percentages of respondents were able to identify sandflies as the transmitting agent (20% in Sudan and 21–88% in the Indian Subcontinent [15], [17]). Also in the urban area of Libo Kemkem knowledge regarding causes was higher and more specific, as 68% of interviewee reported sandflies as the VL causal agent [19].

As might be expected, knowledge regarding preventive measures was also higher in Addis Zemen town, where 20% of respondents identified bed-nets as a VL protection measure, compared to 12% of respondents in the rural communities of our study. Notwithstanding the knowledge regarding bed-net protection is poor in the area as a whole compared to the levels of knowledge found in other studies conducted in Sudan and in the Indian Subcontinent (30–50%) [15], [16], [31]. On the other hand, spraying was barely mentioned in our study which seems to be consistent with results from other VL KAP studies [15], [18]. Potential health education activities should deal with the causes of the disease and the existing protecting measures, which should lead to behavioral changes in the population. It is worth to highlight that in relation to attitudes the level of acceptance for all the protection measures suggested (impregnated bed nets, environmental sanitation and household spraying) was over 94%. However, we are aware that this figure can be overestimated by a possible information bias regarding the health professional's nature of the interviewers who may be known to the community and influence interviewees responses.

The main source of VL knowledge reported in the 2009 survey was the health center, followed by VL patients. Moreover our results show that in May 2009 HH with a positive history of VL in the family had better knowledge overall compared to HH with no history of VL, suggesting that VL patients played an important role as sources of knowledge. This was further supported by the fact that in the post-intervention survey the differences in knowledge between HH with VL positive history and HH with no history of VL practically disappeared, as there was an external source of knowledge common to all the participant families. VL patients, friends and relatives have been identified in other studies as main sources of knowledge, and it is recommended to take the opportunity to appropriately inform and educate them, so they can act as community health agents when they are back into their places of residence [13], [16], [31].

Other health education interventions like broadcasted radio programs have been identified as successful VL information sources [16] but may not be appropriate in this context, where barely a third of the HH own radio. On the contrary, it seems that informative actions, like the ones carried out by the research project presented here, may have a positive impact in the improvement of the disease global knowledge. Our results show that proper knowledge on VL signs and symptoms, on VL causes and on VL protective measures increased by 11, 8 and 8 times respectively between 2009 and 2011. The study design did not allow for comparison with control communities in order to monitor other external factors that could be influencing HH knowledge related to VL. However, we are aware that since the 2005 outbreak, there have been numerous community interventions including outreach activities with health education and cases screening activities. Therefore we believe that the important change in knowledge showed by our results may be a result of the participation of the households in the research project as well as the contribution of all the actors in the area during the study period, emphasizing the potential of indirect education activities in the improvement of VL knowledge in this particular context.

The interpretation of the change in practices observed during the study period is of a different nature, as it is directly related to the malaria prevention and control campaign carried out in the study area during 2009/2010 [32]. This campaign included massive distribution of bed nets and spraying, which accounts for the significant increase in the number of bed nets and in the number of sprayed households [33]. However, although the majority of the HH surveyed owned at least one bed net in 2011, its acceptability as a VL protective tool needs to be ascertained, as a few proportion of the respondents related it to VL protection. Furthermore, in this study we identified that more than 60% of HH had at least one family member sleeping outside, and only a third of those reported to use any protection. They constitute and important group of hosts susceptible to sand fly bites, that need to be aware that sleeping outside without taking appropriate personal vector control measures exposes them to VL infection.

Finally, in 2011, public health facilities were the first choice of treatment in the area (also in Addis Zemen town) [19]. This is probably owed to the quality and free-of-charge nature of health services provided at the VL treatment center located in Addis Zemen Health Center, managed by MSF-Greece during the outbreak, and supported by the Amhara Region Health Bureau and the WHO Leishmaniasis Control Program thereafter. Other studies have shown contrasting results in VL treatment choices, and have associated them with geographical accessibility, treatment costs, confidence in service providers and perceived staff attitude [15], [31], [34]. Our study yielded very low use (≃1%) of traditional medicine for VL treatment compared to other studies in which local healers were consulted by 20 to 50% of the population [17], [35]. These contrasting results may be due to the quantitative methodology used, that may yield different results than qualitative research approaches [14], [34], and may also be influenced by the possible information bias introduced by interviewers being health workers of the area.

Admittedly, our study had some limitations. One of them is that the random sample of HH was taken among *kebeles* highly affected by VL, and therefore we cannot readily extrapolate our findings to the entire population of Libo Kemkem and Fogera districts. However, we believe that the VL knowledge to be detected among the remaining communities (not so affected by VL) would have been even lower. Another limitation is that in order to compare households with and without past history of VL we stratified according to HH status as reported in May 2009 for both surveys, due to the lack of updated information in February 2011. However, the time period of our study coincided with a low plateau in the transmission of Leishmaniasis in the area [9]. Therefore, we believe that only a small number of HH would have proven misclassified, and the general conclusions of our study would have remained the same. Finally, it is possible that an information bias was introduced by having interviewers that were health professionals of the area. However, we believe that this bias would have affected only to responses related to attitudes, as under our consideration, proper knowledge and the practices observed in the study were not subject to change by the influence of an interviewer.

### Conclusions

The VL knowledge in the rural communities of Libo Kemkem and Fogera districts is globally poor, and it should be improved through community strategies. Recommendations are: 1) to conduct sensitization talks in the affected communities, 2) to instruct VL patients and relatives while their stay in the hospital so they can act as health agents in their communities and 3) to follow up the maintenance of bed nets and the use of any other prevention measure like household spraying or environmental sanitation as the high level of acceptance perceived suggests that changes in behaviour are possible.

## Supporting Information

Checklist S1 STROBE checklist.
(DOC)

## References

[1] WHO (2010) Control of the Leishmaniases. Report of a meeting of the WHO Expert Committee on the Control of Leishmaniases.

[2] Alvar J, Velez ID, Bern C, Herrero M, Desjeux P, Cano J, Jannin J, den BM (2012) Leishmaniasis worldwide and global estimates of its incidence. PLoS One 7: e35671. 22693548 10.1371/journal.pone.0035671 PMC3365071

[3] Adhikari SR, Supakankunti S, Khan MM (2010) Incidence of kala-azar in Nepal: estimating the effects of individual and household characteristics. Trans R? Soc Trop Med Hyg 104: 720–725 S0035-9203(10)00187-2 [pii];10.1016/j.trstmh.2010.08.013 [doi] 20875906 10.1016/j.trstmh.2010.08.013

[4] Ali A, Ashford RW (1994) Visceral leishmaniasis in Ethiopia. IV. Prevalence, incidence and relation of infection to disease in an endemic area. Ann Trop Med Parasitol 88: 289–293. 7944674 10.1080/00034983.1994.11812869

[5] Ayele T, Ali A (1984) The distribution of visceral leishmaniasis in Ethiopia. Am? J? Trop Med Hyg 33: 548–552. 6476199 10.4269/ajtmh.1984.33.548

[6] Hailu A, Berhe N, Sisay Z, Abraham I, Medhin G (1996) Seroepidemiological and leishmanin skin test surveys of visceral leishmaniasis in south and southwest Ethiopia. Ethiop Med? J 34: 11–23. 8674496

[7] Herrero M, Orfanos G, Argaw D, Mulugeta A, Aparicio P, Parreno F, Bernal O, Rubens D, Pedraza J, Lima MA, Flevaud L, Palma PP, Bashaye S, Alvar J, Bern C (2009) Natural history of a visceral leishmaniasis outbreak in highland Ethiopia. Am? J? Trop Med Hyg 81: 373–377. 19706898

[8] Bashaye S, Nombela N, Argaw D, Mulugeta A, Herrero M, Nieto J, Chicharro C, Canavate C, Aparicio P, Velez ID, Alvar J, Bern C (2009) Risk factors for visceral leishmaniasis in a new epidemic site in Amhara Region, Ethiopia. Am? J? Trop Med Hyg 81: 34–39. 19556563

[9] Sordo L, Gadisa E, Custodio E, Cruz I, Simon F, Abraham Z, Moreno J, Aseffa A, Tsegaye H, Nieto J, Chicharro C, Canavate C (2012) Low prevalence of Leishmania infection in post-epidemic areas of Libo Kemkem, Ethiopia. Am? J? Trop Med Hyg 86: 955–958 86/6/955 [pii];10.4269/ajtmh.2012.11-0436 [doi] 22665599 10.4269/ajtmh.2012.11-0436 PMC3366538

[10] Wijeyaratne PM, Arsenault LK, Murphy CJ (1994) Endemic disease and development: the leishmaniases. Acta Trop 56: 349–364. 8023758 10.1016/0001-706x(94)90106-6

[11] Custodio E, Gadisa E, Sordo L, Cruz I, Moreno J, Nieto J, Chicharro C, Aseffa A, Abraham Z, Hailu T, Canavate C (2012) Factors associated with Leishmania asymptomatic infection: results from a cross-sectional survey in highland northern Ethiopia. PLoS Negl Trop Dis 6: e1813 10.1371/journal.pntd.0001813 [doi];PNTD-D-11-01209 [pii] 23029576 10.1371/journal.pntd.0001813 PMC3459849

[12] Borges BK, Silva JA, Haddad JP, Moreira EC, Magalhaes DF, Ribeiro LM, Fiuza VO (2008) [Assessment of knowledge and preventive attitudes concerning visceral leishmaniasis in Belo Horizonte, Minas Gerais State, Brazil]. Cad Saude Publica 24: 777–784 S0102-311X2008000400007 [pii]. 18392354 10.1590/s0102-311x2008000400007

[13] Gama MEA, Barbosa JS, Pires B, Cunha AKB, Freitas AR, Ribeiro IR, Costa JML (1998) Evaluation of the level of knowledge about visceral leishmaniasis in endemic areas of Maranhao, brazil. Cad Saude Publica 14: 381–390. 9658223 10.1590/s0102-311x1998000200022

[14] Ahluwalia IB, Bern C, Costa C, Akter T, Chowdhury R, Ali M, Alam D, Kenah E, Amann J, Islam M, Wagatsuma Y, Haque R, Breiman RF, Maguire JH (2003) Visceral leishmaniasis: consequences of a neglected disease in a Bangladeshi community. Am? J? Trop Med Hyg 69: 624–628. 14740879

[15] El Sayed SM, Ahmed SE (2001) Socio-cultural aspects of Kala-azar among Masalit and Hawsa tribes. Ahfad Journal 18: 51.

[16] Koirala S, Parija SC, Karki P, Das ML (1998) Knowledge, attitudes, and practices about kala-azar and its sandfly vector in rural communities of Nepal. Bull World Health Organ 76: 485–490. 9868839 PMC2305780

[17] Mondal D, Singh SP, Kumar N, Joshi A, Sundar S, Das P, Siddhivinayak H, Kroeger A, Boelaert M (2009) Visceral leishmaniasis elimination programme in India, Bangladesh, and Nepal: reshaping the case finding/case management strategy. PLoS Negl Trop Dis 3: e355. 19159009 10.1371/journal.pntd.0000355 PMC2607537

[18] Singh SP, Reddy DC, Mishra RN, Sundar S (2006) Knowledge, attitude, and practices related to Kala-azar in a rural area of Bihar state, India. Am? J? Trop Med Hyg 75: 505–508 75/3/505 [pii]. 16968930

[19] Alemu A, Alemu A, Esmael N, Dessie Y, Hamdu K, Mathewos B, Birhan W (2013) Knowledge, attitude and practices related to visceral leishmaniasis among residents in Addis Zemen town, South Gondar, Northwest Ethiopia. BMC Public Health 13: 382. 23617595 10.1186/1471-2458-13-382 PMC3642032

[20] Federal Democratic Republic of Ethiopia (FDRE), Ministry of Finance and Economic Development (MoFED) (2002) Sustainable Development & Poverty Reduction Program.

[21] Esteso SC (1984) [Popular education–a fragile point in the campaign against Chagas' disease]. Rev Fac Cien Med Univ Nac Cordoba 42: 14–17. 6443701

[22] da Luz ZM, Pimenta DN, Rabello A, Schall V (2003) Evaluation of informative materials on leishmaniasis distributed in Brazil: criteria and basis for the production and improvement of health education materials. Cad Saude Publica 19: 561–569. 12764472 10.1590/s0102-311x2003000200023

[23] de Magalhaes DF, da Silva JA, Haddad JP, Moreira EC, Fonseca MI, de Ornelas ML, Borges BK, da Luz ZM (2009) Dissemination of information on visceral leishmaniasis from schoolchildren to their families: a sustainable model for controlling the disease. Cad Saude Publica 25: 1642–1646 S0102-311X2009000700025 [pii]. 19578588 10.1590/s0102-311x2009000700025

[24] Luz ZM, Schall V, Rabello A (2005) Evaluation of a pamphlet on visceral leishmaniasis as a tool for providing disease information to healthcare professionals and laypersons. Cad Saude Publica 21: 606–621 S0102-311X2005000200028 [pii];/S0102-311X2005000200028 [doi] 15905924 10.1590/s0102-311x2005000200028

[25] Nieves E, Villarreal N, Rondon M, Sanchez M, Carrero J (2008) [Evaluation of knowledge and practice on tegumentary leishmaniasis in an endemic area of Venezuela]. Biomedica 28: 347–356 S0120-41572008000300005 [pii]. 19034358

[26] Alvar J, Bashaye S, Argaw D, Cruz I, Aparicio P, Kassa A, Orfanos G, Parreno F, Babaniyi O, Gudeta N, Canavate C, Bern C (2007) Kala-azar outbreak in Libo Kemkem, Ethiopia: epidemiologic and parasitologic assessment. Am? J? Trop Med Hyg 77: 275–282. 17690399

[27] Gadisa E, Custodio E, Canavate C, Sordo L, Abebe Z, Nieto J, Chicharro C, Aseffa A, Yamuah L, Engers H, Moreno J, Cruz I (2012) Usefulness of the rK39-immunochromatographic test, direct agglutination test, and leishmanin skin test for detecting asymptomatic Leishmania infection in children in a new visceral leishmaniasis focus in Amhara State, Ethiopia. Am? J? Trop Med Hyg 86: 792–798. 22556076 10.4269/ajtmh.2012.11-0196 PMC3335682

[28] Hailu A, Musa AM, Royce C, Wasunna M (2005) Visceral leishmaniasis: new health tools are needed. PLoS Med 2: e211 05-PLME-ND-0101 [pii];10.1371/journal.pmed.0020211 [doi] 16033309 10.1371/journal.pmed.0020211 PMC1181879

[29] WHO-WHA (2007) Control of leishmaniasis. Sixtieth World Health Assembly WHA60.13.

[30] Mondal D, Alam MS, Karim Z, Haque R, Boelaert M, Kroeger A (2008) Present situation of vector-control management in Bangladesh: a wake up call. Health Policy 87: 369–376 S0168-8510(08)00034-1 [pii];10.1016/j.healthpol.2008.01.011 [doi] 18342389 10.1016/j.healthpol.2008.01.011

[31] Siddiqui NA, Kumar N, Ranjan A, Pandey K, Das VN, Verma RB, Das P (2010) Awareness about kala-azar disease and related preventive attitudes and practices in a highly endemic rural area of India. Southeast Asian? J? Trop Med Public Health 41: 1–12. 20578475

[32] Ministry of Finance and Economic Development (MoFED) (2010) Ethiopia: 2010 MDGs Report:Trends and Prospects for Meeting MDGs by 2015.

[33] Ministry of Health (2010) Health Sector Development Program IV 2010/11-2014/15.

[34] Gerstl S, Amsalu R, Ritmeijer K (2006) Accessibility of diagnostic and treatment centres for visceral leishmaniasis in Gedaref State, northern Sudan. Trop Med Int Health 11: 167–175 TMI1550 [pii];10.1111/j.1365-3156.2005.01550.x [doi] 16451340 10.1111/j.1365-3156.2005.01550.x

[35] Kolaczinski JH, Hope A, Ruiz JA, Rumunu J, Richer M, Seaman J (2008) Kala-azar epidemiology and control, southern Sudan. Emerg Infect Dis 14: 664–666. 18394290 10.3201/eid1404.071099 PMC2570907

